# Resilience and regulation, an odd couple? Consequences of Safety-II on governmental regulation of healthcare quality

**DOI:** 10.1136/bmjqs-2019-010610

**Published:** 2020-03-30

**Authors:** Ian Leistikow, Roland A Bal

**Affiliations:** 1 Dutch Health & Youth Care Inspectorate, Utrecht, The Netherlands; 2 Erasmus School of Health Policy & Management, Erasmus University, Rotterdam, The Netherlands

**Keywords:** patient safety, risk management, health policy, root cause analysis

## Introduction

The concept of resilience, or Safety-II, is finding its way into national patient safety policies. In the Netherlands, for example, hospital, professional and patient federations have named Safety-II as one of the three pillars for the new national patient safety strategy.^1^ In July 2019, National Health Service (NHS) England and NHS Improvement published the NHS Patient Safety Strategy which also strives to embed Safety-II principles in the national policy.^2^ National policies of any kind require a form of public accountability, and for quality and safety in healthcare this accountability is mostly regulated by external, often governmental, regulatory authorities. However, while most current research on Safety-II addresses activities of front-line workers and clinical leadership, the role of external regulatory systems is hardly addressed.^3^ The relationship between regulation and Safety-II and the role regulators could play in improving or undermining Safety-II performance, needs investigation and theorising.^4 5^ In this article, we combine theory with practice examples to show how Safety-II principles could influence the interactions between healthcare providers and their regulators.

## The core concepts of Safety-II

In general, Safety-II is about learning from things that go right and improving resilience, where Safety-I is about learning from things that go wrong and improving compliance. The five core concepts of Safety-II are:

1. Definition of safety: ‘Safety’ is not defined as the absence of failures or adverse outcomes, which is considered ‘Safety-I thinking’, but as the ability to make things go right.^6^
2. Safety management: Safety management is focused on maintaining adaptive capacity to respond effectively to inevitable surprises.^7^
3. Role of humans: Humans are not seen as a risk, but as a resource necessary for system flexibility and resilience.^7^
4. Accident investigation: The purpose of accident investigations is to understand how things usually go right, since that is the basis for explaining how things occasionally go wrong.^7^
5. Risk assessment: Risk assessment is focused on understanding ‘conditions where performance variability can become difficult or impossible to monitor and control’.^7^

In summary, Safety-II is about learning from things that go right, with a focus on ‘Work As Done’ (WAD) instead of ‘Work As Imagined’ (WAI), meaning the paper-based reality of rules and guidelines. Safety-II is not intended as a replacement, but as a complement to Safety-I. The founding fathers of the theory state that the two perspectives on safety must co-exist, at least for the foreseeable future.^6^

## The core concepts of regulation

Regulation is in some countries referred to as supervision, scrutiny, oversight or inspection. In this article we will use the term regulation, which is classically defined as control exercised by a public agency over activities which are valued by a community.^8^ Although simplification does too little justice to the vast body of scholarly work on regulation, basically, regulation has three objectives^9^:

- To improve performance and quality.
- To provide assurance that minimally acceptable standards are achieved.
- To provide accountability for levels of performance.

How regulators are meant to do this, is an issue of ongoing debate. The past decades have seen a shift from compliance-based regulation towards responsive and reflexive regulation.^10 11^ This development, in which inspectors focus less on rules and paperwork and more on context and interaction with regulatees, can be seen as somewhat similar to the development of Safety-II thinking. However, the extent to which regulators make this shift differs per sector. Regulation in healthcare is complex and involves a range of agencies using different approaches, often dependent on their legal mandate. Regulation of pharmaceutical products, for example, is grounded in international legislation which gives the national regulators little room to tailor their interventions. In regulation of elderly care or hospitals, national regulators have much more leeway to develop strategies fitting to the local values and cultures. A common issue in all sectors is that governmental regulators need to account for their actions. How both patients, healthcare professionals, politicians and the general public perceive these actions, determines the legitimacy and societal value of governmental healthcare regulators.^12^

## Where regulation meets resilience

Regulation in healthcare is often associated with compliance to rules and guidelines, which on face value would seem the antithesis of Safety-II. But the concepts of regulation and Safety-II are actually quite similar; both are about making sense of situations in the context of their social dynamics.^4^ The five core concepts of Safety-II can help understand how regulation and Safety-II influence each other (see table 1).

**Table 1:** The five core concepts of Safety-II, their consequences for accountability and regulation, and examples

Theme	Safety-II concept for the theme	Consequences for accountability of healthcare providers	Consequences for regulation	Current examples
Definition of safety	‘Safety’ entails that as many things as possible go right	Providers will have to report on improvements in the number of things that go right, and on underlying argument on what is ‘right’	Providers and regulators need to agree on what is ‘right’ and how this relates to ‘Work As Done’	Regulators’ use of the Short Observational Framework for Inspection as method for inspectors to assess the quality of care for people with dementia
Safety management principle	Proactive; continuously trying to anticipate developments and events	Providers should show they have structures and processes in place with which to respond effectively to unforeseen situations	Regulators will use conversations with boards and inspections on site to assess how consistent the boards stories are with experiences on shop floor	Regulation of care for the disabled through format-free Quality Reports that providers publish
The human factor in safety management	Humans are seen as a resource necessary for system flexibility and resilience. Humans provide flexible solutions to many potential problems	Focus on (interdisciplinary) teamwork, accessibility of higher management for healthcare professionals’ experiences and ‘Joy in work’	Regulators should engage in open conversation with healthcare providers on how they empower their employees to fulfil this role adequately	Requirement for ‘peer support’ after serious adverse events
Accident investigation	The purpose of an investigation is to understand how things usually go right as a basis for explaining how things occasionally go wrong	External accountability will also require healthcare providers to look into what went wrong	Regulators could combine Safety-I and Safety-II by judging whether the healthcare provider has looked into why the event occurred and into what could be done to strengthen resilience	Healthcare providers using ‘functional resonance analysis method’ to analyse adverse events
Risk assessment	Focused on understanding the conditions where performance variability can become difficult or impossible to monitor and control	Providers should report on how the organisation monitors and controls performance variability	Regulators can stimulate or mandate systems that monitor performance variability	Regulators assessing whether providers use ‘Quality Registries’

1. Definition of safety: if ‘safety’ is defined as the ability to make things go right, a dialogue will be needed on what ‘right’ is and how healthcare providers can account for their level of performance in ‘getting things right’. This is harder than it seems. While ‘wrong’ is often easily established by the occurrence of unintended harm, the absence of harm does not mean that care has ‘gone right’. Moreover, there are often different ‘rights’ in play and healthcare providers need to compromise between those ‘rights’. Abiding by guidelines, for example, does not automatically entail providing the ‘right’ care, just as driving a car without creating an accident is in itself no measure of how safe one drives. Also, a surgeon’s definition of ‘right’ could be different from a patient’s. Patient expertise has already been said to offer opportunities for resilience in healthcare,^13^ and this will be essential in defining what ‘right’ is with regard to healthcare quality.
  The regulatory regime could focus on these two elements.
  - How has the healthcare provider defined what it considers ‘right’ and how has it engaged the four perspectives (patient, professional, politics, public) in this consideration.
  - Does the healthcare provider achieve minimum levels of ‘right’ and how does it account for its performance in relation to WAD.
    An example of a regulatory strategy in this regard is the use of the Short Observational Framework for Inspection (SOFI) as method for inspectors to assess the quality of care for people with dementia by observing WAD.^14^ Regulators as the English Care Quality Commission (CQC), Scottish Care Inspectorate and Dutch Health and Youth Care Inspectorate are using SOFI to this end.
2. Safety management: If healthcare providers want to move away from rigid guidelines and towards adaptive capacity, they will have to find a way to show that they have the structures and processes in place with which to respond effectively to unforeseen situations. This will require inspectors to engage in narrative conversation with healthcare providers on how they have organised their safety management and follow these up with inspections on site to assess how the narrative resonates with WAD.
  An example of how this could work is seen in regulation of care for the disabled by the Dutch Health and Youth Care Inspectorate. Agreement between the main stakeholders led to a change in the national policy, shifting away from preset indicators and quality measures towards narrative accountability.^15^ Care providers now submit an annual Quality Report which has no specified format. These are assessed by inspectors and used as starting point for open conversations with care providers. Interestingly, several of these care providers are starting to create guidelines for these Quality Reports, suggesting unease with the lack of structure in their process of accountability.
3. Role of humans: If humans are expected to be a resource necessary for system flexibility and resilience, then the work environment will need to enable employees to fulfil this role. This requires a focus on (interdisciplinary) teamwork, easy accessibility of higher management for healthcare professionals’ quality concerns and ‘joy in work’. Employees will need to be alert enough to recognise things going wrong and empowered to act or speak up.
  An example of how hospitals organise these elements is through the introduction of ‘crew resource management’.^16^ Regulatory oversight would require healthcare providers to account for how they empower their employees to be the resource as intended.
  An example of a regulatory strategy that relates to this theme, is the requirement of the Dutch Health and Youth Care Inspectorate to provide a form of ‘peer support’ for employees who were involved in serious adverse events. This contributed to an increase of peer support being mentioned in adverse event investigation reports from 40% in 2013 to 100% by 2016 (based on internal data from the Dutch Health and Youth Care Inspectorate).
4. Accident investigation: of the five core concepts of Safety-II, this one seems the least compatible with the core concepts of regulation. When serious unintended harm occurs, patients, public and politicians often expect answers to what happened, why it happened and what is being done to prevent recurrence. An investigation exclusively focused on how things usually go right, will most likely be interpreted as deflecting responsibility and lead to calls for disciplinary action. Here we feel Safety-I and Safety-II could be combined. Take, for example, a case in which a fatal diagnostic delay occurred because an ultrasound was ordered but not executed. A Safety-I investigation would look into why the ultrasound was not executed, while a Safety-II investigation would look into why ordering an ultrasounds in similar situations usually goes right. Several healthcare organisations in countries like Denmark, Australia and the Netherlands are experimenting with Functional Resonance Analysis Method (FRAM), the investigation method based on Safety-II thinking.^17^ FRAM tries to identify how healthcare processes usually take place, and define measures for improving resilience. Combining FRAM with a Safety-I investigation could lead to a higher level of learning and align the Safety-II and regulatory goals.
5. Risk assessment: Monitoring and understanding everyday performance variability has proven to be very demanding for healthcare providers. Quality registries are an example of how this can be organised. Inspired by Sweden, many countries have engaged in setting up registries that monitor and try to understand the everyday performance for specific diseases and treatments, mostly for acute hospital care. In the Netherlands, the Dutch Institute for Clinical Audits (DICA) supports 22 of these quality registries and reports annually on quality improvements that these have helped realise. Setting up and maintaining a quality registry is resource intensive and requires commitment from frontline personnel to fill in the required data. In 2019, the Dutch Parkinson registry was abandoned because the administrative burden was too high and poor reporting led to outcomes that could not be used to improve the quality of care or help patients in their choice for a care provider.^18^
  The Dutch Health and Youth Care Inspectorate has helped the DICA gain traction by relating several national hospital quality indicators to DICA registries. This, for example, led to a nationwide adoption of the Dutch Surgical Colorectal Audit within 2 years. The switch from requiring hospitals to have morbidity and mortality rounds, to requiring hospitals to include their patients in national quality registries, can be seen as an example of how regulation can switch from Safety-I to Safety-II.

## Conclusion

Although the positive language of Safety-II is appealing, actually enacting the principles will be a challenge to both healthcare providers and their regulators. It will require a certain maturity from each actor, internally and in its relationship with other actors. The regulatory focus will shift from compliance to consistency: how does a provider’s WAI as presented by board and management play out in WAD as enacted in everyday performance? A gap between WAI and WAD is known as ‘decoupling’.^19^ Earlier studies have shown how regulators can support the reverse process of recoupling in regulated organisations by changing the focus from prescriptive regulation based on quality and safety indicators to supervision of the management system.^20^ One could call this a move from regulatory oversight to regulatory insight. The central challenge for regulators will be to give healthcare professionals the required level of freedom to tailor quality management to their local conditions, while simultaneously retaining trust from patient, politics and public that the regulator will intervene timely and effectively when quality falls short and healthcare providers fail to learn from their mistakes. In practice this means regulation can probably never be premised exclusively on Safety-II.

Nonetheless, regulation has the capacity to actively support Safety-II development, and can provide mechanisms and structures through which resilience is generated across healthcare systems.^4^ The goals of Safety-II are also in line with those of a public regulator. We therefore feel that regulation and Safety-II are not the odd couple they might seem, and could be at the beginning of a great friendship.
